# Analgesic and Functional Efficiency of High-Voltage Electrical Stimulation in Patients with Lateral Epicondylitis—A Report with a 180-Day Follow-Up

**DOI:** 10.3390/jcm11092571

**Published:** 2022-05-04

**Authors:** Paweł T. Dolibog, Beata Porębska, Sławomir Grzegorczyn, Daria Chmielewska, Andrzej Ślęzak, Patrycja Dolibog

**Affiliations:** 1 Department of Biophysics, Faculty of Medical Sciences in Zabrze, Medical University of Silesia, 19 H. Jordan Str., 41-808 Zabrze, Silesia, Poland; grzegorczyn@sum.edu.pl; 2 Department of Neurology with Stroke Subdepartment, Provincial Hospital in Bielsko-Biała, 101 Armii Krajowej Avenue, 43-316 Bielsko-Biała, Silesia, Poland; beaps@wp.pl; 3 Electromyography and Pelvic Floor Muscles Laboratory, Department of Physical Medicine, Institute of Physiotherapy and Health Sciences, The Jerzy Kukuczka Academy of Physical Education, 72a Mikołowska Str., 40-065 Katowice, Silesia, Poland; d.chmielewska@awf.katowice.pl; 4 Department of Health Science, Jan Dlugosz University, 13/15 Armia Krajowa Al., 42-200 Częstochowa, Silesia, Poland; aslezak52@gmail.com; 5 Department of Medical Biophysics, Faculty of Medical Sciences in Katowice, Medical University of Silesia, 18 Medyków Str., 40-752 Katowice, Silesia, Poland; pdolibog@sum.edu.pl

**Keywords:** lateral epicondylitis, tennis elbow, high-voltage electrical stimulation, pain intensity, visual analogue scale, hand grip strength

## Abstract

The available publications describing the beneficial effects of electrostimulation does not unequivocally confirm the clinical utility of high-voltage electrical stimulation (HVES) in the treatment of the lateral epicondylitis (LE). The aim of this study was the estimation of the effect of HVES on pain intensity and functional efficiency, both in the short and long term in patients with LE. The trial was registered by the Australian and New Zealand Clinical Trials Registry (ACTRN12621001389897). There were 58 patients allocated into two groups: the HVES group (n = 29, mean age 49.9 ± 11.0 years), treated with HVES (pulse duration: 200μs, frequency: 100 Hz, current amplitude in the range of 18–25 mA, voltage amplitude: 100 V), and the NORM group (*n* = 29, mean age 48.0 ± 12.6 years), who were healthy and untreated patients. The treatments were performed 5 days a week (from Monday to Friday) for two weeks. Treatment progress was measured by the visual analogue scale (VAS) for rest pain, night pain, and pain during activity; the Laitinen Pain Scale (LPS); and hand grip strength (HGS) before and after the treatment, as well as after 3, 6, 12, and 24 weeks. The reduction of pain (according to the VAS and LPS) and increase in the functional condition (according to the HGS) were observed in all HVES patients in the short- and long-term observation. Therefore, the HVES in treatment of LE was found to be effective and safe.

## 1. Introduction

Treatment of the lateral epicondylitis (LE), also known as a tennis elbow (TE), can be divided into conservative treatment, mainly aimed at reducing pain, and surgical treatment, when conservative therapy is unsuccessful. LE is manifested most often by pain in the elbow area and a reduced in grip strength. It affects 1–3% of the adult human population between the ages of 40 and 60 [1,2,3,4,5,6,7].

The fast development of medical devices, the low cost of treatments, and rarely occurring side effects are the reason that physical methods are more often used in the treatment of enthesopathy of the lateral humeral epicondylitis, which shortens the time of treatment and can be used instead of pharmacological treatment or even surgical treatment [2,3,4,5,6,7,8].

The effectiveness of physical methods was confirmed by a large number of research works using, e.g., high- and low-intensity laser therapy (HILT and LILT), shock wave therapy (SWT), ultrasound therapy (UD), kinesiotaping (KT), and electrostimulation (ES). The review of the literature on the treatment of LE shows great interest in shock wave therapy (SWT), and there are also reports in the literature on the use of UD, LILT, and KT [8]. Researchers showed high effectiveness of using shock wave therapy and compared it with effectiveness of LILT [2], KT [3], and UD [4,5]. Moreover, also used are a combination of therapies combined with a hot pack, ES and EWST, or UD or KT [6].

To assess the outcome of therapy, researchers most often use the VAS scale, grip strength tests, and various functional tests: the Duruöz Hand Index (DHI) [5], Patient-Rated Tennis Elbow Evaluation (PRTEE) [2,5], Roles and Maudsley Score (RMS) [3,4], and Disability of the Arm Shoulder and Hand Questionnaire (DASH) [2,3]. All studies show a significant difference of the results obtained in the questionnaires for assessing the effectiveness of therapy.

**Figure 1:**
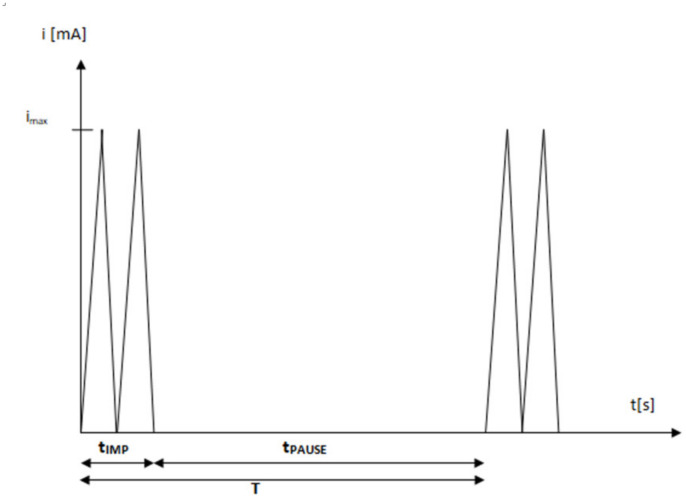
The typical characteristics of waveform in HVES (pulse duration: t_IMP_ = 0.01 T; pause duration: t_PAUSE_ = 0.99 T; period: T = t_IMP_ + t_PAUSE_; duty cycle: t_IMP_/T.).

High-voltage electrostimulation (HVES) is a therapeutic current consisting of a monophasic double impulse (there are single- and two-phase impulses), the voltage of which is usually in the range of 100–500 V. The pulse duration is between 5 and 200 µs, and the frequency is in the range from 1 to 125 Hz. The duration of the double pulse is about 1% of the period, while the pause time is 99% of the period. Hence, the duty cycle factor can be determined, i.e., the ratio of the pulse duration to the pulse period, which is equal to 0.01 for this waveform (Figure 1) [9,10,11,12,13,14]. The waveform with such parameters allows for deep penetration of the muscle fibers, as well as stimulation of sensory and motor nerve fibers and skeletal muscles. The electric charge accumulated in the tissues is neutralized in the interruptions in the impulses by the tissue homeostatic mechanisms. The short duration of the pulse eliminates the possibility of tissue damage and allows for the use of high-amplitude voltage. Due to the low current values, electrophysiological effects are more visible in tissues than electrochemical ones. HVES causes direct depolarization of cell membranes. Under the influence of HVES, motor and sensory fibers of deeply located nerves may be recruited. The short duration of the impulse does not allow the Aδ and C sensory fibers to be stimulated, which results in minimal pain sensations [15].

In general, the availability of publications describing the beneficial effects of electrostimulation in the treatment of tennis elbow is low. Currently, there are a few scientific papers that describe the use of electrostimulation in most cases, in combination with other physical therapy in LE. However, these trials do not include the effect of HVES in the TE problem.

Radpasand et al. [16] used HVES to treat chronic TE (selected parameters: 150 Hz, for 10 s duration and current amplitude in the range from 19 to 29 mA, depending on the participant’s tolerance and counterforce bracing, ice, and exercises for four patients). The results of treatment were matched with the results of treatment of another two patients being treated with UD, counterforce bracing, and exercise. Despite the fact that the authors focus on testing the protocol in the pilot study and not on specific values of the measured parameters, they indicate the effectiveness of the analgesic effect and improve the pain-free grip strength test in both combined multimodal therapies used.

Nourbakhsh et al. [17] used noxious-level electrical stimulation (4 Hz, interrupted DC current, during six treatment sessions over a 2–3-week period) and placebo to treat chronic TE. The authors concluded that the electrostimulation used in the study in tender places has a significant analgesic effect, improves grip strength, reduces activity due to pain, and improves functional abilities in people with chronic lateral epicondylitis.

Chesterton et al. [18] presented in detail the design, methods, and use of TENS (frequency 110 Hz, pulse duration 200 µs, and intensity individually adjusted to the pain threshold, performed daily for 6 weeks and lasting 45 min) in the treatment of LE only as design of the study and not a presentation of the research results.

A similar study was presented by Wend et al. [19]. They investigated the effect of TENS (current frequency of 5 kHz) modulated in two frequency ranges (with a modulation of 2–4 Hz, while in group II, with a frequency of 100 Hz) administered with acupuncture needles. Group III was the control group. The treatments lasted 20 min and were performed three times a week for 2 weeks. Patients were assessed with the VAS scale before the start of the study and after 2 weeks. This work demonstrated the beneficial effect of low- and high-frequency currents in pain relief.

Połtawski et al. tried to find the optimal parameters of microcurrent therapy in reducing pain intensity, improving the function and strength of the clamp. In the first trial, they had two groups of patients and used a monophasic current of 50 and 500 µA for 3 weeks, once a day for 99 min for 21 days. The second trial was also divided into two groups, for the first one, a monophasic current of 40 µA once a day for 6 h and for 3 weeks was applied, which gave the total duration of treatments 189 h. Moreover, in the second group, a regulated current of 40–500 µA was applied for three weeks, except that in the first week, it was applied three times a day for 30 min for 5 days; in the second week, twice a day for 30 min for 5 days; and in the third week, once a day for 30 min for 5 days (15 h in total). The authors performed assessments after 3, 6, and 15 weeks of treatment. The best results were obtained in the first trial for the first group, where a monophasic current and a constant intensity of 50 µA were used (93% of patients felt much better and were healed). These results were maintained 15 weeks after the end of treatment [20].

Full methodological descriptions, electrical parameters, inclusion and exclusion criteria, randomization, and analysis of early later results in cited manuscripts are not always provided.

Therefore, the present study was proposed with the objective of evaluating the effects of HVES on pain and range of hand grip strength in LE.

The purpose of this study was to investigate the efficacy of high-voltage electrical stimulation in patients with chronic lateral epicondylitis of the humerus. The effectiveness of HVES use was assessed by examining pain intensity (according to the VAS and LPS scale) and functional efficiency (according to the HGS), both in the short and long term.

## 2. Materials and Methods

### 2.1. Study Design

**Figure 2:**
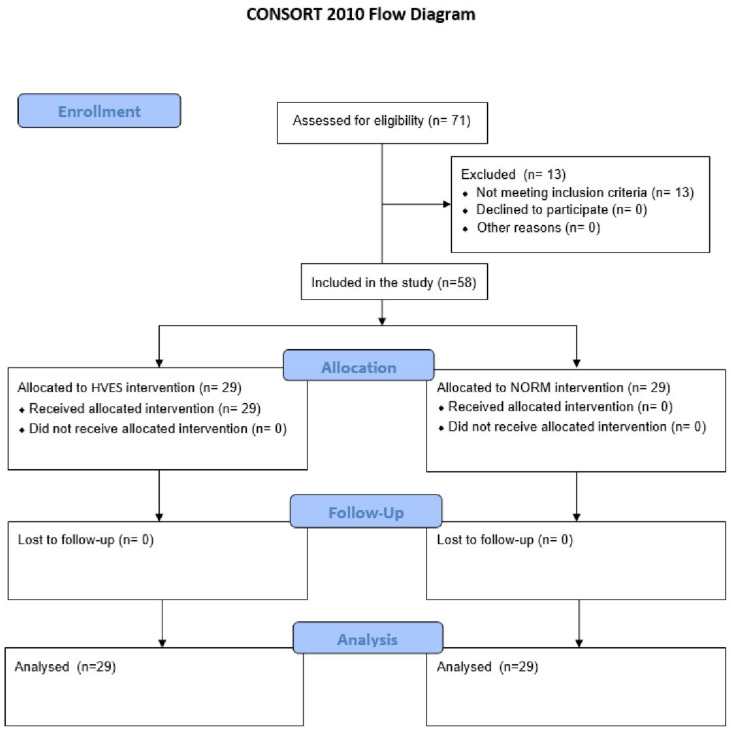
The CONSORT 2010 flow diagram of patients in the study. Abbreviations: HVES, high-voltage electrical stimulation; NORM, control group.

A single-center (Pain Treatment Clinic of the Beskid Oncology Center—City Hospital in Bielsko-Biała, Poland) study was conducted from December 2014 to November 2016. The study was approved by the Bioethics Committee of Bioethics Commission of the Medical University of Silesia in Katowice, Poland (KNW/0022/KB1/158/10). All patients provided signed informed consent for this project. All clinical investigation was conducted according to the principles in the Declaration of Helsinki. The study was performed according to the requirements of the Consolidated Standards of Reporting Trials (CONSORT) statement (Figure 2) [21]. The trial is registered in the Australian and New Zealand Clinical Trials Registry (ACTRN12621001389897).

### 2.2. Participants

In the study, 58 adult outpatients (34 females and 24 males) participated, with a mean age of 48.9 ± 11.7 years (range: 20–72) and a median of 51 years (quartile range: 40–58). Patients were placed into two comparative groups: HVES (*n* = 29, 17 females and 12 males) with a mean age of 49.9 ± 11.0 years, and NORM (*n* = 29, 17 females and 12 males) with a mean age of 48.0 ± 12.6 years. The average duration of disorder in the HVES group was 12.7 ± 15.6 with a median of 4 months (range: 1–70). In the NORM group, the patients did not suffer from chronic lateral epicondylitis (LE) of the humerus. Patients from the comparative groups were homogeneous in terms of gender, age, height, weight, and BMI (Table 1).

### 2.3. Sample Size

On the basis of the results of the pilot study, we estimated the sample size necessary to demonstrate a statistical significance of *p* = 0.05 and a test power of 0.8 within the group scores. The resulting minimum sample size was 24 patients in the HVES group. The target sample size was 29. The sample size analysis was performed using Statistica (TIBCO Software Inc., Palo Alto, USA, 2017).

### 2.4. Qualification

#### 2.4.1. HVES Group

We included patients with LE qualified by a neurologist, a radiologist, and an orthopedist in order to confirm the disease. Each patient had an X-ray examination of the elbow joint in two anterior–posterior (AP) and lateral projections before the procedures were started. Patients with permanent pain in the lateral epicondyle for more than one month in one upper limb were qualified for the study.

The exclusion criteria for patients in the HVES group were as follows: cervical and thoracic spinal pains, fractures, golfer’s elbow, pregnancy, having a pacemaker, rheumatic dis-eases, cancer, cardiovascular diseases, metal implants, corticosteroid injections carried out within the last 6 weeks, diabetes, skin inflammation, and being under 18 or over 75 years of age.

#### 2.4.2. NORM Group

Healthy persons were enrolled in the NORM group by a neurologist. All patients had a physical and medical examination. We included patients with lack of pain and correct mobility in the upper limb. All persons subjected to examination and measurements were in good general condition, not requiring therapeutic intervention for any other reason.

The exclusion criteria for patients in NORM group were the same as in the HVES group.

### 2.5. Interventions

In the HVES group, all the treatments were performed with the Ionoson Physiomed PMD–91220 high-voltage electrical generator (Physiomed Elektromedizin AG, Schnaittach, Germany).

**Figure 3:**
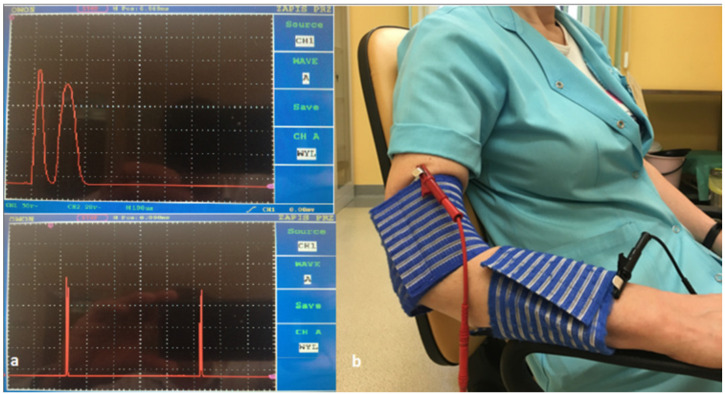
(**a**) Therapeutic current applied in therapy. (**b**) Placement of electrodes during therapy.

Before performing each individual procedure for a patient, the stimulation current was checked for the generated electric current waveform in a 10 kΩ resistor connected in parallel with the generator (Ionoson Physiomed PMD–91220) and the oscilloscope (OWON PDS7102T- Fujian Lilliput Optoelectronics Technology Co., Ltd., Zhangzhou, China) (Figure 3a).

The current waveform of treatment consisted of double monophasic pulses with a total duration of 200 µs and a frequency of 100 Hz, with a voltage amplitude of 100 V. Regulation of the root mean square current of the generator was from 18 to 25 mA (average 21.5 mA) and was adjusted individually to each patient depending on their sensitivity to pain tolerance. The stimulation was performed with a current that did not cause any motor effects, only a tingling sensation. A conductive silicone electrode (Physiomed Elektromedizin) with dimensions of 8 × 6 cm and an area of 48 cm² was used. The active electrode (anode) was placed on the lateral epicondyle of the diseased upper limb. The passive electrode (cathode) was placed on the forearm of the sick upper limb. The electrodes were placed in a viscose casing soaked in physiological saline (Figure 3b).

The treatments were performed 5 days a week (from Monday to Friday), every day for a period of two weeks. Each treatment lasted 50 min. The procedure was performed in a sitting position, the patient’s arm was abducted, the elbow joint was bent at a 60-degree angle, the forearm was pronated, and the arm and forearm were supported by the chair’s armrest (Figure 3b).

In the NORM group, no physical treatments and stabilization training were applied. Hand grip strength was measured at the same control intervals as for the HVES group.

### 2.6. Outcome Measurements

The therapeutic outcome measurement progress of the Hydraulic Hand Dynamometer Analogue (HHDA, SH5001 Saehan Corporation, Masan, Korea), the VAS analogue scale, and the Laitinen Pain Scale (LPS) were used before treatments; immediately after treatment; and a 3, 6, 12, and 24, weeks follow-up.

The HHDA was used to analyze an average hand grip strength parameter, including the strength of the flexor muscles of the fingers of the hand, resistance flexion in the wrist, and resistance extension in the wrist.

The VAS was used to measure level of rest pain, night pain, and physical activity pain. The score of 0 was assigned for no pain, and the score of 10 was assigned for the strongest pain.

The LPS was used to assess 4 indicators: pain intensity, frequency of pain occurrence, use of analgesics, and limitations of mobility. It contains four areas to be assessed on a scale from 0 to 4, where the number 0 means no problem in a given area, and the number 4 means its maximum degree. The maximum score for four indicators is 16 points, with the lower the LPS score, the better it is for the patient.

### 2.7. Statistical Analysis

The normality of the distribution of the data was tested by means of the Shapiro–Wilk test. To compare variables in both groups of patients, a chi-squared test of independence (the highest level of reliability) and the Mann–Whitney U test were used. To compare the measured values (strength, pain in the VAS scale, and LPS) obtained before; immediately after; and at 3 weeks, 6 weeks, 12 weeks, and 24 weeks after therapy with the HVES, the non-parametric Friedmann ANOVA test and post hoc Dunn–Bonferroni test were used.

The reliability hand muscle strength was assessed by the intraclass correlation coefficient (ICC). ICC values and their 95% confidence intervals were calculated on the basis of a values of six Hydraulic Hand Dynamometer Analogue recordings [22].

All statistical analyses were performed using the STATISTICA software (TIBCO Software Inc., Palo Alto, CA, USA, 2017) and PQStat software (PQStat Software, Poznań, Polnad). The level of statistical significance was assumed at *p* = 0.05.

## 3. Results

In total, 58 patients were enrolled. All patients were monitored for 180 days after therapy. The treatment was effective for all patients.

### 3.1. Analysis of Results Regarding the Influence of High-Voltage Electrical Stimulation Therapy on the Subjective Experience of Functional Ability in Patients with Elbow Pain

The Friedmann ANOVA analysis of variance indicated that changes of hand grip strength in the group treated with HVES were statistically significant (*p* < 0.001), but post-hoc comparisons showed that there were no statistically significant differences (0.103) only before and immediately after the study. Moreover, after 3, 6, 12, and 24 weeks after therapy, the differences in hand grip strength were statistically significant (Table 2).

The study of resistance wrist flexion (Table 3) and resistance extension (Table 4) in the wrist showed that both the results of the variance analysis and the post hoc tests were statistically significant. In both cases, there was an increase in strength both immediately after the test and in the following weeks (3, 6, 12, 24).

The reliability of measurements of the hand grip strength, resistance wrist flexion strength, and resistance extension in the wrist strength in the NORM group was checked using the intraclass correlation coefficient (ICC). The analysis carried out for the measurements of the hand grip strength showed a significant agreement of the measurements (*p* < 0.001); the reliability of the measurement was ICC = 0.991 for six time intervals. Similar reliability results were obtained in resistance wrist flexion strength (*p* < 0.001, ICC = 0.987) and resistance extension in the wrist strength (*p* < 0.001, ICC = 0.991) measurements.

### 3.2. Analysis of Influence of HVES Therapy on the Subjective Experience of Elbow Pain

**Figure 4:**
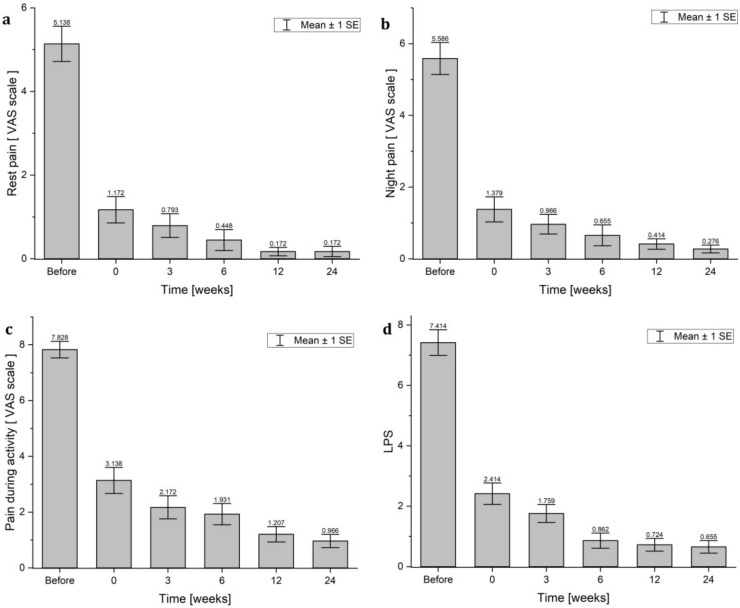
The comparison of changes in VAS points: (**a**) rest pain; (**b**) night pain; (**c**) pain during activity) and (**d**) LPS scores in the HVES group: before treatment; 0—after treatment; and 3, 6, 12, and 24 weeks after study completion.

Directly after the end of the treatments for patients with HVES treatment, a statistically significant rest pain reduction was observed, with a median from 5 points (range 0–10) before to 0 points (range 0–7) after treatment (*p* < 0.001) on the VAS scale. Rest pain was gradually reduced to 0 points (range 0–3) 24 weeks after treatment (*p* < 0.001) (Table 5 and Figure 4a). The same dependence was observed in the reduction of the value of night pain (Table 4 and Figure 4b) and pain during activity (Table 4 and Figure 4c). In both cases, the analysis of variance showed the statistically significant reduction of night pain and pain during activity (*p* < 0.001). Night pain and pain during activity were gradually reduced. Interestingly, the maximal reduction of pain (rest, night, and activity) was observed immediately after treatment (statistically significant differences) (Table 5).

The situation was very similar for the assessment of the LPS (Table 4 and Figure 4d). The Friedmann ANOVA analysis of variance showed that changes of LPS score in the group treated with HVES were statistically significant (*p* < 0.001). The LPS median value decreased from 7 points (range 4–12) before treatment to the value of 2 points (range 0–9) after treatment (*p* = 0.002). Moreover, in 3, 6, 12, and 24 weeks, the LPS values were gradually decreased in time to 0 points (range 0–4).

It is important that we do not see any deterioration in the hand grip strength, VAS scale, and LPS scale values in long-term observations. Moreover, significant statistical decrease during long-time follow-up indicated a positive analgesic effect of HVES therapy.

## 4. Discussion

The analgesic effect of electricity was known and used in the treatment of many diseases, mainly those related to back pain, e.g., lower back pain [23,24]. The main reasons for using electricity as an analgesic therapy are the lack of side effects, the relatively low cost of the procedure, and the widespread availability of the therapy [25]. Our novel use of HVES resulted in reduction of pain (VAS) and improved mobility range and function efficiency (LPS) of the affected limb just after treatment and follow-up observations.

In the available literature, several studies confirm the analgesic effects of electric current in treatment of LE [16,17,18,19,20].

There are no scientific reports on the effectiveness of HVES in the treatment of LE with the exception of one. Nevertheless, Radpasand et al. [16] used HVES but in conjunction with counterforce bracing, ice, and exercises (only in four patients), and therefore the effect of HVES on the treatment of tennis elbow cannot be independently assessed. Thus, this is the first study that shows the effectiveness of treating LE with HVES.

The electric current waveform used in this protocol was established on the basis of experience in the use of the analgesic effect of the current. In various reports [16,17,18,19,20], the duration of the procedure with the use of electricity varies, and there is no strictly defined time, nor number of treatments.

In our study, we used twin-peak monophasic electric current waveform with very short pulse duration of 200 μs, frequency of 100 Hz, and voltage amplitude of 100 V. The current intensity was used in the range from 18 to 25 mA (average 21.5 mA) at the level adapted to the patient’s tolerance. In our study, we used rubber electrodes with a surface area of 48 cm^2^ (6 × 8 cm). Patients were treated for 50 min per visit for 2 weeks from Monday to Friday (in total, 500 min).

Radpasand et al. [16] used HVES—150 Hz, for 10 s duration and current in the range from 19 to 29 mA—for the participants’ tolerance. Nourbakhsh et al. [17] used a noxious level of electrical stimulation at 4 Hz and interrupted DC current for six treatment sessions over a 2–3-week period. Chesterton et al. [18] used transcutaneous electrical stimulation of TENS: 110 Hz, pulse duration of 200 µs, and current intensity individually adjusted to the pain threshold. The treatments were performed daily for 6 weeks and lasted 45 min. Wend et al. [19] investigated the effect of TENS modulated in two frequency ranges: 5 kHz with a modulation of 2–4 Hz, and 100 Hz. The treatments lasted 20 min and were performed three times a week for 2 weeks. Połtawski et al. [20] used a monophasic current of 50 and 500 µA for 3 weeks, once a day for 99 min for 21 days, and monophasic current of 40 µA once a day for 6 h and for 3 weeks, which gave the total duration of treatments 189 h. In the second group, a regulated current in the range of 40–500 µA was applied for three weeks.

There are differences between our parameters of the applied current for electrostimulation and those used by other scientists. The frequency of the current closest to the one used by us is noted in Radpasand et al. [16] and Chesterton et al. [18] and in one of the stimulations by Wend et al. [19]. The current intensity used by us was also very similar to the scientific work by Radpasand et al. [16].

A single procedure lasted from 20 min to 6 h, and the entire treatment took up to 3 weeks (usually 3–5 times a week). This is similar to our protocol.

The results of pain reduction in our research are consistent with other scientific reports on the use of electrostimulation in reducing pain. It can be seen that after 2 weeks of using pain relief therapy (immediately after treatment), there was a significant reduction in the value of rest pain, night pain, and pain during activity.

In all the works cited by us, the analgesic effect of electrostimulation was observed. For subjective pain assessment, Radpasand et al. [16] and Wend et al. [19] used the VAS scale, while Nourbakhsh et al. [17] used the numeric rating scale of the NRS, which is the same as the VAS scale. Połtawski et al. [20] did not use a scale to assess pain; however, they did report pain reduction after electrostimulation. An analgesic effect has been observed after treatment and a prolonged effect has also been observed. We investigated the analgesic effect by means of VAS scale: rest pain, night pain, and pain during activity. Our results and those reported by other scientists are similar.

A review of the literature shows that the researchers present hand strength results as a promising tool. Radpasand et al. [16], Połtawski et al. [20], and Nourbakhsh et al. [17] used the pain-free grip strength PFGS protocol to assess the strength of the hand grip. In our study, the following parameters were measured: dynamometric hand grip strength, dynamometric wrist flexor strength, and dynamometric wrist extensor strength. Both our team and another scientist observed an improvement in the hand grip strength. There were no differences between our results and those of other scientists.

All studies show a significant difference of the results obtained in the questionnaires for assessing the effectiveness of therapy. Patient-rated LE evaluation PRTEE was used to assess pain and functionality of patients with tennis elbow (Radpasand et al. [16], Nourbakhsh et al. [17], Wend et al. [19], Połtawski et al. [20]). We used the LPS scale, which also assesses pain and functionality in patients, but it is a more universal tool as it can be used regardless of the disease entity. Both in the PRTEE bag and in the LPS scale, we observed a relationship between the obtained points and the feeling of pain and functionality. Less points means less pain and better functionality. The results obtained by us are consistent with the results of the cited articles publications.

The objective of this trial was to find a safe, cost-effective, and available primary care intervention for LE. Our research and analysis of the results demonstrate that the use of HVES is an effective tool in the treatment of tennis elbow because it reduces pain; improves functionality; and increases hand grip strength, wrist flexor strength, and wrist extensor strength. Moreover, it has been proven that the use of monotherapy provides healing effects on a similar level to combined treatment. In addition, it is a therapy based on widely available devices, is relatively cheap, and has no side effects.

We are aware of the limitations of the work, which should be noted. We did not compare other known treatments for tennis elbow enthesopathy in our study because we wanted to demonstrate the usefulness of the HVES method in the treatment of pain and look at the long-term effects. At a later stage, we want to compare the method with a focused shock wave, UD, and other electrical therapies (including TENS or Bernard’s currents) and answer to the question: which method of treatment is more effective?

## 5. Conclusions

HVES treatment reduces pain and increases functional conditions in relation to baseline. The HVES method is effective and safe for patients with LE, which was confirmed by short- and long-term observations.

## Figures and Tables

**Table 1:** Demographic and anthropometric characteristics of patients in HVES and NORM groups. *p*—Mann–Whitney U test; *p* *—χ^2^ (chi-squared) test. SD—standard deviation.

Parameter	HVES Group	NORM Group	*p*-Value
Total (*n*)	29	29	
Gender—female/male (*n*)	17/12	17/12	1 *
Age (years)			
Mean	49.9	48.0	
SD	11.0	12.6	
Median	51.0	50.0	0.769
Range	29.0–72.0	20.0–65.0	
Height (m)			
Mean	168.8	168.0	
SD	9.1	7.7	
Median	167.0	168.0	0.781
Range	153.0–182.0	150.0–184.0	
Weight (kg)			
Mean	76.4	74.5	
SD	16.1	15.5	
Median	75.0	70.0	0.665
Range	49.0–110.0	55.0–106.0	
Body weight status by World Health Organization standards			
Normal weight (%)	12 (41)	12 (41)	
Overweight (%)	13 (45)	9 (31)	0.352 *
Obesity (%)	4 (14)	8 (28)	
Affected side			
Right	19	-	-
Left	10		

**Table 2:** The dynamometric measurement of the hand grip strength. ICC, interclass correlation coefficient model (for six trials of the hand grip strength). Abbreviations: W0, baseline (before treatment); W1, after treatment; W2, 3 weeks after treatment; W3, 6 weeks after treatment; W4, 12 weeks after treatment; W5, 24 weeks after treatment.

Group	W0	W1	W2	W3	W4	W5	*p* Friedmann ANOVA **, Dunn–Bonferroni * (against the Baseline) Test
HVES (kG)							Main effect, *p* ** < 0.001
Mean	24.1	30.9	34.2	36.4	36.9	37.1	W0 vs. W1, *p* * = 0.103
SD	13.4	12.9	12.7	11.9	11.8	12.4	W0 vs. W2, *p* * < 0.001
Median	22.7	29.0	31.7	31.7	31.7	31.7	W0 vs. W3, *p* * < 0.001
Min	2.3	8.2	13.6	20.4	22.7	20.4	W0 vs. W4, *p* * < 0.001
Max	49.9	54.4	57.6	61.2	57.6	58.9	W0 vs. W5, *p* * < 0.001
NORM (kG)							
Mean	34.3	34.4	34.3	33.6	34.2	34.1	
SD	10.2	9.4	9.4	9.4	9.8	9.5	ICC(2,k) = 0.991
Median	31.7	33.6	31.9	31.3	32.2	32.4	−95% CI ICC(2,k) = 0.988
Min	18.1	19.5	19.1	18.1	17.2	18.1	+95% CI ICC(2,k) = 0.994
Max	61.7	56.7	54.0	56.0	56.0	56.0	*p* < 0.001
*p*—Mann–Whitney U test	<0.001	0.134	0.638	0.534	0.564	0.600	

**Table 3:** The dynamometric measurement of wrist flexor strength. ICC, interclass correlation coefficient model (for six trials of the wrist flexors strength). Abbreviations: W0, baseline (before treatment); W1, after treatment; W2, 3 weeks after treatment; W3, 6 weeks after treatment; W4, 12 weeks after treatment; W5, 24 weeks after treatment.

Group	W0	W1	W2	W3	W4	W5	*p* Friedmann ANOVA **, and Dunn–Bonferroni * (against the Baseline) Test
HVES (kG)							Main effect, *p* ** < 0.001
Mean	6.8	9.0	9.6	10.2	10.9	10.8	W0 vs. W1, *p* * = 0.038
SD	3.4	3.8	4.3	4.6	4.0	4.0	W0 vs. W2, *p* * < 0.001
Median	6.8	8.4	8.8	9.6	10.7	9.3	W0 vs. W3, *p* * < 0.001
Min	1.7	4.5	4.5	4.5	4.5	4.5	W0 vs. W4, *p* * < 0.001
Max	12.7	17.2	18.1	20.4	20.4	20.4	W0 vs. W5, *p* * < 0.001
NORM (kG)							
Mean	9.9	9.8	9.9	9.9	9.7	9.8	
SD	3.5	3.6	3.7	3.9	3.6	3.6	ICC(2,k) = 0.987
Median	10.0	10.0	9.9	9.7	9.7	9.7	−95% CI ICC(2,k) = 0.980
Min	4.5	4.1	4.5	3.6	4.1	4.5	+95% CI ICC(2,k) = 0.991
Max	18.1	17.2	18.0	22.0	18.0	18.0	*p* < 0.001
*p—* Mann–Whitney U test	<0.001	0.154	0.505	0.968	0.249	0.465	

**Table 4:** The dynamometric measurement of wrist extensor strength. ICC, interclass correlation coefficient model (for six trials of the wrist extensors strength). Abbreviations: W0, baseline (before treatment); W1, after treatment; W2, 3 weeks after treatment; W3, 6 weeks after treatment; W4, 12 weeks after treatment; W5, 24 weeks after treatment.

Group	W0	W1	W2	W3	W4	W5	*p* Friedmann ANOVA **, and Dunn–Bonferroni * (against the Baseline) Test
HVES (kG)							Main effect, *p* ** < 0.001
Mean	4.1	5.9	6.7	7.4	7.7	7.8	W0 vs. W1, *p* * = 0.038
SD	2.9	3.3	3.7	3.9	3.7	3.8	W0 vs. W2, *p* * < 0.001
Median	3.4	4.5	4.5	5.4	7.0	7.5	W0 vs. W3, *p* * < 0.001
Min	0.4	2.3	2.3	3.2	3.6	2.3	W0 vs. W4, *p* * < 0.001
Max	10.4	13.6	13.6	15.9	15.9	15.9	W0 vs. W5, *p* * < 0.001
NORM (kG)							
Mean	6.6	6.7	6.5	6.5	6.6	6.6	
SD	2.6	2.6	2.2	2.3	2.4	2.3	ICC(2,k) = 0.991
Median	6.0	6.4	6.0	6.0	6.6	6.6	−95% CI for ICC(2,k) = 0.986
Min	2.7	2.7	2.3	2.3	2.3	2.3	+95% CI for ICC(2,k) = 0.993
Max	13.6	13.6	10.9	10.9	10.9	11.3	*p* < 0.001
*p*—Mann–Whitney U test	<0.001	0.049	0.493	0.989	0.510	0.313	

The HGS for patients in the NORM group (34.3 ± 10.2 kG; 336.4 ± 100.0 N) were statistically significantly greater than the HGS in the patients in the HVES group (24.1 ± 13.4 kG; 236.3 ± 131.4 N) only before treatment (*p* < 0.001). However, in long-term observations, the hand grip strength in the HVES group was slowly increased, and 3 weeks after therapy was at the level of value in the NORM group (34.2 vs. 34.3 kG; 335.4 vs. 336.4 N), and further gradually increased up to 24 weeks after treatment (37.1 vs. 34.1 kG; 368.8 vs. 334.4 N).

**Table 5:** The VAS and LPS results of within-group HVES at treatment (points). Abbreviations: W0, baseline (before treatment); W1, after treatment; W2, 3 weeks after treatment; W3, 6 weeks after treatment; W4, 12 weeks after treatment; W5, 24 weeks after treatment.

Parameter (Points)	HVES Group	W0	W1	W2	W3	W4	W5	*p* Friedmann Annova **. and Dunn Bonferoni * Test
The VAS results: rest pain	Mean SD Median Min Max	5.1 2.3 5.0 0.0 10.0	1.2 1.7 0.0 0.0 7.0	0.8 1.5 0.0 0.0 6.0	0.4 1.3 0.0 0.0 6.0	0.2 0.5 0.0 0.0 2.0	0.2 0.7 0.0 0.0 3.0	Main effect, *p* ** < 0.001 W0 vs. W1, *p* * < 0.001 W0 vs. W2, *p* * < 0.001 W0 vs. W3, *p* < 0.001 W0 vs. W4, *p* * < 0.001 W0 vs. W5, *p* * < 0.001
The VAS results: night pain	Mean SD Median Min Max	5.6 2.4 6.0 0.0 10.0	1.4 1.9 1.0 0.0 6.0	0.9 1.5 0.0 0.0 5.0	0.7 1.6 0.0 0.0 8.0	0.4 0.8 0.0 0.0 3.0	0.3 0.6 0.0 0.0 2.0	Main effect, *p* ** < 0.001 W0 vs. W1, *p* * < 0.001 W0 vs. W2, *p* * < 0.001 W0 vs. W3, *p* * < 0.001 W0 vs. W4, *p* * < 0.001 W0 vs. W5, *p* * < 0.001
The VAS results: pain during activity	Mean SD Median Min Max	7.8 1.6 8.0 3.0 10.0	3.1 2.5 3.0 0.0 9.0	2.2 2.2 2.0 0.0 8.0	1.9 2.1 2.0 0.0 8.0	1.2 1.5 1.0 0.0 5.0	0.9 1.3 1.0 0.0 4.0	Main effect, *p* ** < 0.001 W0 vs. W1, *p* * = 0.001 W0 vs. W2, *p* * < 0.001 W0 vs. W3, *p* * < 0.001 W0 vs. W4, *p* * < 0.001 W0 vs. W5, *p* * < 0.001
The LPS results	Mean SD Median Min Max	7.4 2.3 7.0 4.0 12.0	2.4 1.9 2.0 0.0 9.0	1.8 1.6 2.0 0.0 6.0	0.9 1.4 0.0 0.0 5.0	0.7 1.1 0.0 0.0 4.0	0.7 1.1 0.0 0.0 4.0	Main effect, *p* ** < 0.001 W0 vs. W1, *p* * = 0.002 W0 vs. W2, *p* * < 0.001 W0 vs. W3, *p* * < 0.001 W0 vs. W4, *p* * < 0.001 W0 vs. W5, *p* * < 0.001

## Data Availability

The data presented in this study are available on request from the corresponding author.
